# Identifying, Assessing, and Supporting Family Caregivers in Health and Long-Term Care: Progress and Opportunities

**DOI:** 10.1093/geroni/igab046.1036

**Published:** 2021-12-17

**Authors:** Catherine Riffin, Jennifer Wolff

**Affiliations:** 1 Weill Cornell Medicine, New York, New York, United States; 2 Johns Hopkins Bloomberg School of Public Health, Baltimore, Maryland, United States

## Abstract

Family caregivers are a largely hidden but vital workforce within medical and long-term care settings. Family caregivers are actively involved throughout care delivery systems and provide crucial assistance to people with chronic conditions. Building on the person- and family-centered care approach and recent recommendations from national organizations, this presentation sets forth a roadmap for research, policy, and practice that outlines practical solutions and opportunities to address existing barriers to systematic assessment and support of family caregivers in clinical practice. With the impending family care gap and projections for a steep decline in the availability of family caregivers in the coming decades, it is more important than ever to prepare health care systems for this shift. If put into action, the recommendations of this presentation can help to bridge the care gap by promoting sustainable solutions and infrastructure to ensure that families are recognized and adequately supported in care delivery settings.

